# Tibial plateau fractures in elderly people: an institutional retrospective study

**DOI:** 10.1186/s13018-018-0986-8

**Published:** 2018-11-01

**Authors:** Qi-fang He, Hui Sun, Lin-yuan Shu, Yu Zhan, Chun-yan He, Yi Zhu, Bin-bin Zhang, Cong-feng Luo

**Affiliations:** 1 Department of Orthopaedic Surgery, Shanghai Jiao Tong University Affiliated Sixth People’s Hospital, 600 YiShan Road, Shanghai, 200233 China; 2 Department of Emergency, Shanghai Jiao Tong University Affiliated Sixth People’s Hospital, 600 YiShan Road, Shanghai, 200233 China; 3 Chongqing Health Center for Women and Children, 64 Jintang Street, Yuzhong District, Chongqing, 400013 China

**Keywords:** Classification, Epidemiology, Geriatric fractures, Morphology, Tibial plateau fractures

## Abstract

**Background:**

Tibial plateau fractures are the most common intra-articular fractures, which require careful evaluation and preoperative planning. The treatment of tibial plateau fractures in elderly patients is challenging, and the comprehension of epidemiology and morphology can be helpful. This study described the characteristics of geriatric tibial plateau fractures.

**Methods:**

A total of 327 (23.24%) patients aged ≥60 years were reviewed in our level one trauma center over a 4-year period (from January 2013 to November 2016). The following parameters were collected and evaluated: (1) demographic data, (2) injury mechanisms and (3) fracture classifications.

**Results:**

Females accounted for 60.86% in all included elderly patients. Electric-bike accidents were the cause of 32.42% of all these injuries, and 39.62% of these led to high-energy injuries. The most common type of fracture was Schatzker II (54.74%). According to the three-column classification, single lateral column fracture (28.75%) and four-quadrant fracture (involving lateral, medial, posterolateral and posteromedial fractures) (23.24%) were the two most frequent patterns. In all cases, 67.58% involved the posterior column, and the prevalence of posterolateral and posteromedial fractures were 62.69% and 37.92% respectively. Isolated posterior column fractures accounted for 12.54% of patients in total, which mostly consisted of posterolateral fracture in older females (85.37%).

**Conclusions:**

The majority of elderly patients with tibial plateau fractures are females, and Electric-bike accidents are an important cause of injury. Geriatric tibial plateau fractures have unique distribution in classification.

## Introduction

Tibial plateau fractures (TPFs) are relatively common, accounting for approximately 1% of all fractures, and the population-based incidence of TPFs has been reported as 10.3–13.3 per 100,000 people annually [1, 2]. Cases of TPFs were most common between the ages of 30 years and 60 years [3, 4]. However, with improved life expectancy, incidences of TPFs in elderly patients are probably rising [5, 6]. Comprehension of the epidemiology and morphology can be helpful to manage the fractures, but there is little epidemiological information available and to date virtually none about the morphology of TPFs focusing on the elderly population [6, 7]. This current study reports the basic epidemiology and morphological classification of TPFs in elderly patients in a level one trauma center, including incidence, injury mechanisms, combined injuries, and fracture classifications.

In the literature, the AO/OTA and Schatzker classifications were the most frequently used to assess the morphology and severity of TPFs. Because both classifications provide only two-dimensional information of the fracture, a CT-based three-column classification (TCC) would be a good supplement for evaluation [8]. There were already numerous studies which indicated that implementation of the three-column classification (TCC) might improve the surgical outcome of cases of TPFs [9, 10]. This is the first time that the TCC has been used to evaluate the morphological features of tibial plateau fractures in a large sample size of elderly patients.

## Materials and methods

### Patients

The approval of our institution’s ethical review board was obtained prior to initiation of the study. The study included all patients treated for TPFs over a 4-year period (from January 2013 to November 2016) in the trauma center of our hospital, which is a level one trauma center. Patients were excluded based on the following criteria: (1) isolated avulsion fracture of tibial plateau, such as tibial avulsion fractures of anterior or posterior cruciate ligament, Segond fractures; (2) suspected fractures which could not be confirmed by X-ray radiographs or computerized tomography (CT) scans; (3) fractures in children and skeletally immature adolescents; and (4) pathological or old fractures, namely, the fractures for more than 3 weeks. Finally, 1407 patients with TPFs were included and 327 elderly patients aged ≥ 60 years (23.24%) were isolated for further analysis.

### Epidemiology

The following parameters of patients aged ≥ 60 years were collected and evaluated: (1) demographic data, (2) injury mechanism, (3) combined injuries, and (4) fracture classification. According to local conditions and lifestyles, we subdivided traffic accidents into the car, E-bike, and bicycle accidents and differentiated falls as being from height (more than 2 m), from medium height (less than 2 m), or on the ground. Other causes of injury included industrial and agricultural-related accidents, sport-related, and fighting-induced accidents.

### Fracture classification

**Fig. 1:**
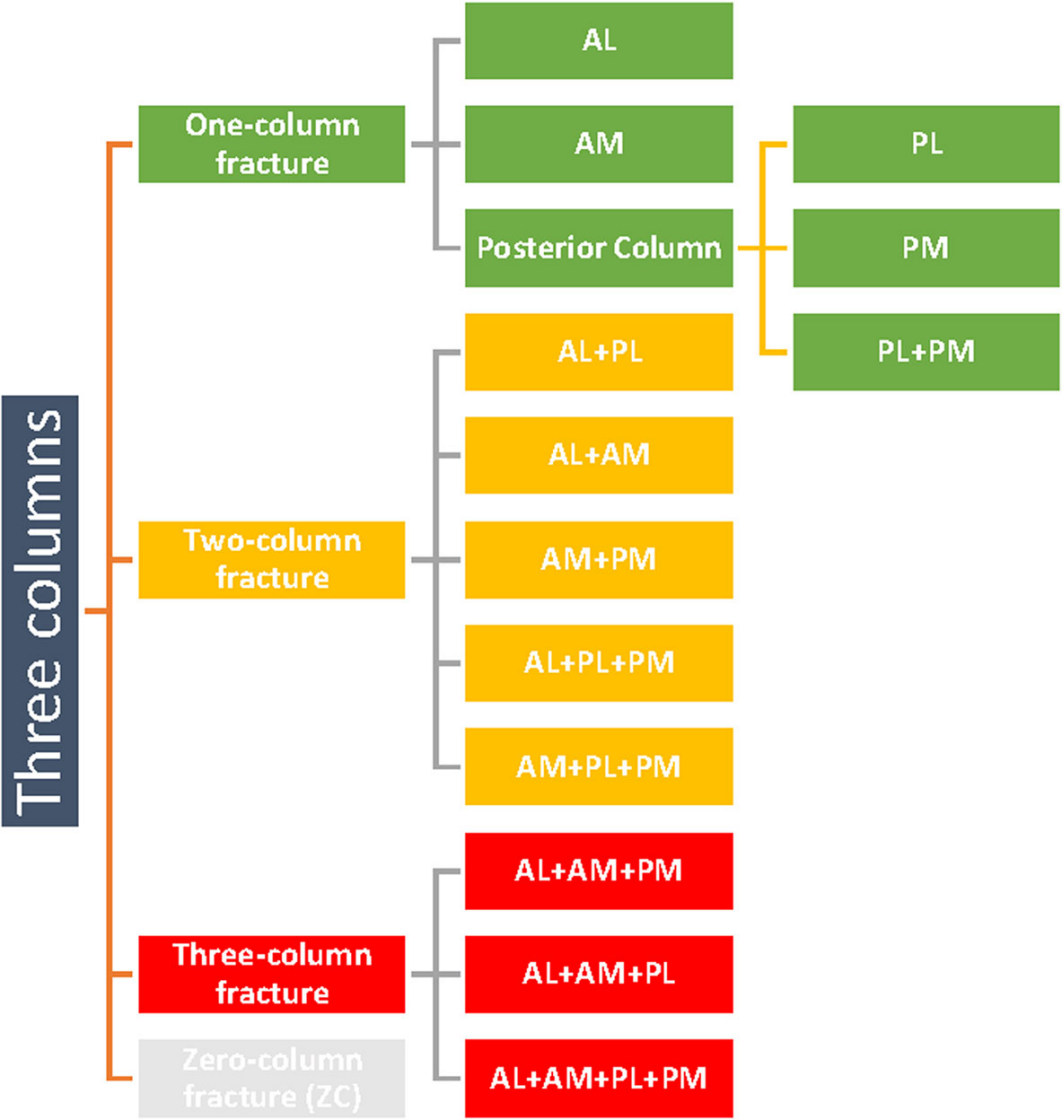
The subdivision of TPFs by TCC. According to the three-column classification (TCC), the tibial plateau is divided into three columns, including the lateral (anterolateral), medial (anteromedial), and posterior columns. The posterior column is then subdivided into two sub-columns, the posterolateral and posteromedial sub-columns. The fractures could be divided into 14 patterns, in which four different quadrants of the tibial plateau might be involved. AL, anterolateral column; AM, anteromedial column; PL, posterolateral sub-column; PM, posteromedial sub-column

The images of normal X-ray and CT scans of enrolled patients were obtained from Picture Archiving and Communication System (PACS) workstations. Both the X-ray-based Schatzker classification and CT-based TCC were all applied to evaluate the fractures’ morphology. Based on the TCC, the tibial plateau is divided into three relatively independent columns, individually defined as the lateral, medial, and posterior columns as observed on the images of the CT axial plane and three-dimensional reconstructions. An articular depression alone without cortical splitting is defined as a “zero-column (ZC)” fracture. The fractures involving the cortex of lateral and medial columns are renamed as anterolateral (AL) and anteromedial (AM) pattern fractures. A fracture involving the cortex of the posterior column is further divided into posterolateral (PL) and posteromedial (PM) pattern fractures. The reason for this division is based on different injury mechanisms and a distinct operation plan including the choice of surgical approaches and fixation strategies [11, 12]. The subdivision of tibial plateau fracture by TCC is summarized in Fig. 1.

The classifications of all fractures were executed by a research team consisting of a chief surgeon, an attending surgeon of orthopedic trauma, and two resident surgeons. The chief surgeon (C-F L) and attending surgeon (Hui S) were the original contributors to the TCC system, and the other surgeons were trained and familiar with both classification systems (the Schatzker classification and TCC). The consensus of classification for each fracture was made among all members of the team to achieve an accurate evaluation.

### Statistics

Continuous variables were presented as the mean and range values, and categorical data as frequencies and percentages. The chi-square test was used to compare the differences between male and female, injury mechanisms, and fracture types. SPSS 19.0 software (Statistical Package for Social Sciences, Inc., Chicago, IL, USA) was used for all analysis. *P* < 0.05 was considered statistically significant.

## Results

### Epidemiology

**Fig. 2:**
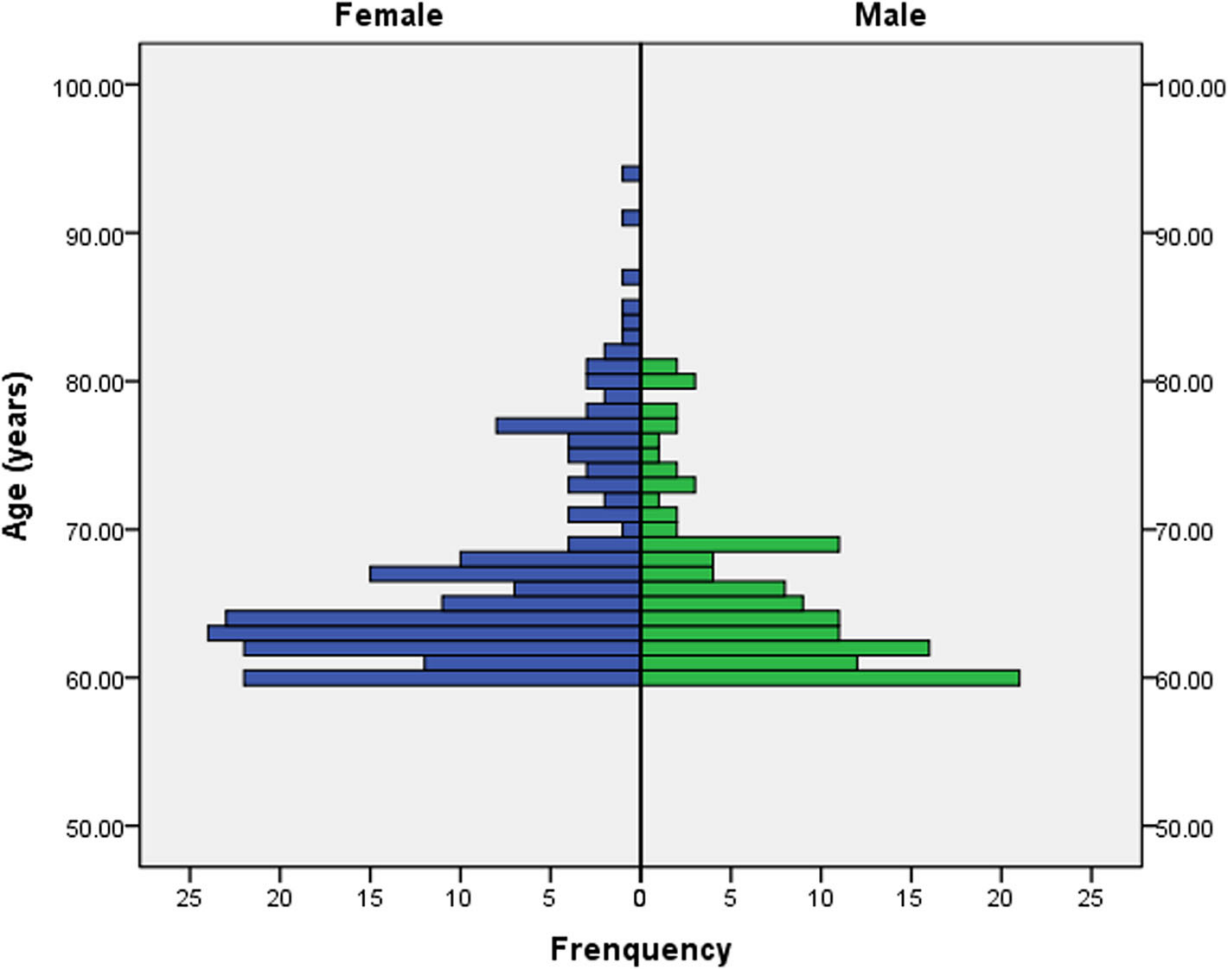
The age distribution of elderly patients with TPFs between females and males. Most of the elderly patients were aged between 60 and 70 years

**Fig. 3:**
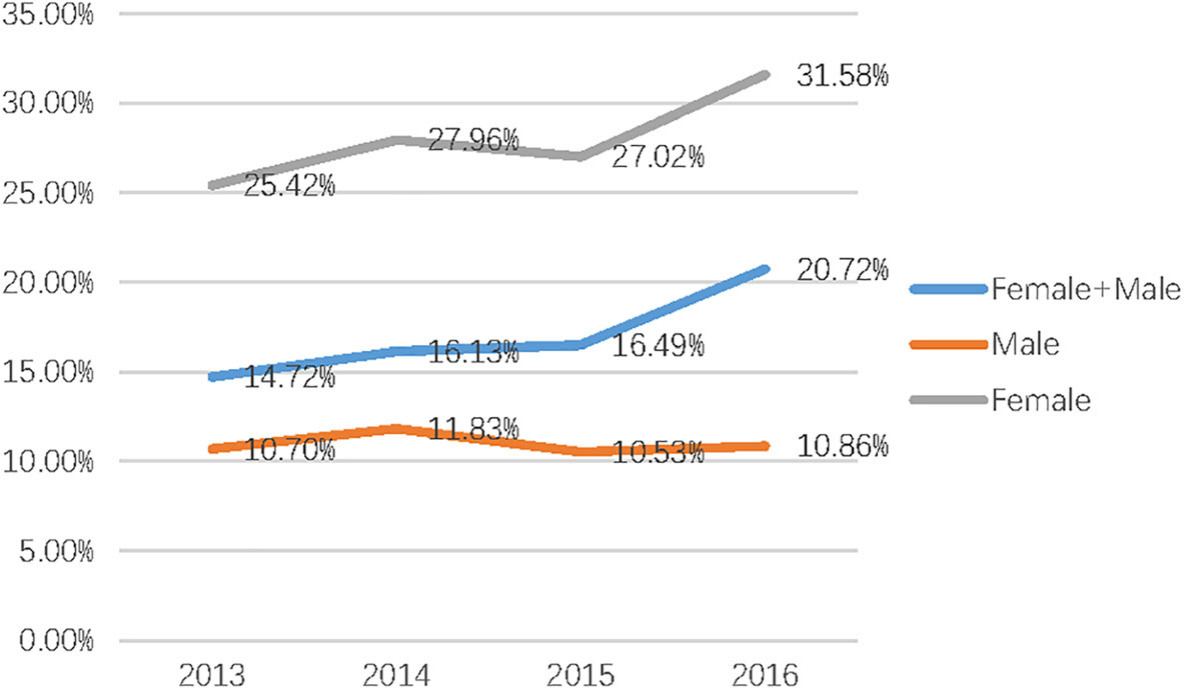
The incidence of geriatric TPFs from 2013 to 2016. The number of elderly patients with TPFs, especially female patients, presented an increasing trend

Most of the elderly patients were aged between 60 and 70 years (Fig. 2). The average age of all elderly patients with TPFs was 66.4 years (range 60–94 years), and males (mean age 65.5 years; range 60–81 years) were younger than females (mean age 67.00 years; range 60–94 years) (*P* = 0.013). Of these patients, 60.86% (199/327) were females and the number of female patients was increasing (Fig. 3). Among the 327 elderly patients, 277 suffered from a single injury, and 37 patients (11.31%) had injuries that were combined with additional injuries. The distal femoral (10 cases) and upper extremity fractures (9 cases) were the most frequently combined injuries. The majority of injury mechanisms were traffic accidents, especially involving an electric bike (E-bike) (106/327, 32.42%) (Table 1).

**Table 1:** Distribution of injury mechanisms

Injury mechanisms	Females	Males	Total (percent)
Traffic injuries			
Car accidents	21	19	40 (12.23%)
E-bike accidents	69	37	106(32.42%)
Bicycle	28	13	41(12.54%)
Fall injuries			
From height	13	22	35(10.7%)
From medium height	42	26	68(20.80%)
On ground	17	7	24(7.34%)
Others	9	4	13(3.98%)

*E-bike* electric-bike

### Schatzker classification

According to the Schatzker classification, type II accounted for more than half of all fractures (179/327, 54.74%), followed by type V (47/327, 14.37%), type VI (45/327,13.76%), type III (29/327, 8.87%), type VI (22/327, 6.73%), and type I (5/327, 1.53%). Schatzker type I TPFs only occurred to males, while females tended to have a higher incidence of Schatzker type II TPFs and a lower incidence of type VI fractures compared with males (*P* < 0.05). There was no significant gender difference in Schatzker type I, III, IV, and V fractures (*P* > 0.05).

**Fig. 4:**
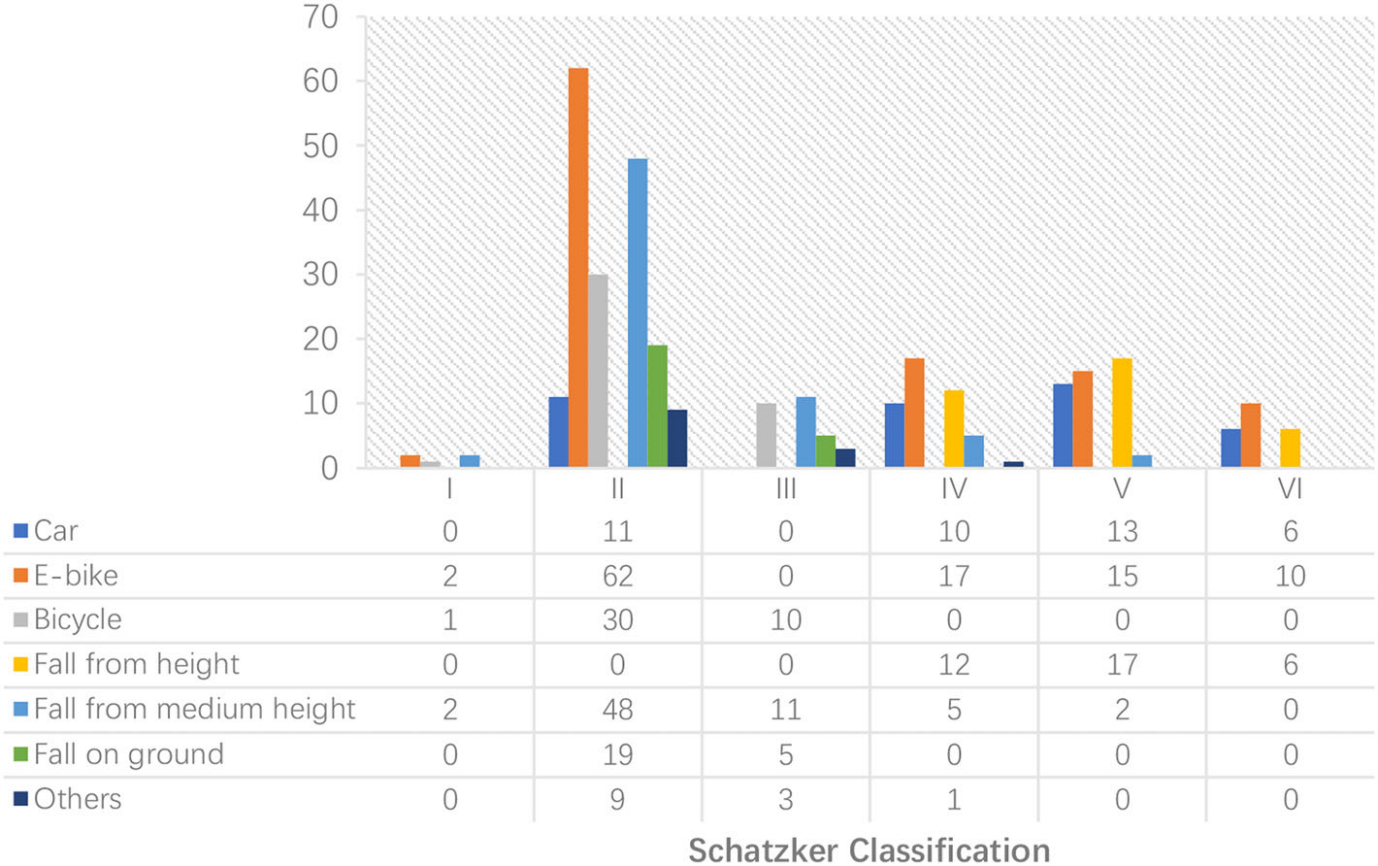
The distribution of injury mechanism according to the Schatzker classification in elderly patients. Females tended to have a higher incidence of Schatzker type II TPFs and a lower incidence of type VI fractures when compared with males. There is no significant gender difference in Schatzker type I, III, IV, and V fractures

The relationship between Schatzker classification and injury mechanism is demonstrated in Fig. 4. Injuries from E-bike use caused 62 cases of Schatzker type II (58.49%) fractures and 42 cases (39.62%) of severe injuries, including Schatzker type IV, V, and VI fractures.

### Three-column classification (TCC)

On the basis of TCC, the incidences of different fracture patterns were analyzed in elderly patients. The one-column fractures were the most commonly occurring injuries (66.67%), followed by the two-column (32.72%), and three-column fractures (23.85%). Compared with male patients, female patients had a higher incidence of one-column fractures (44.2% vs 39.06%, *P* < 0.05) and a lower incidence of three-column fractures (22.61% vs 25.78%, *P* < 0.05).

**Fig. 5:**
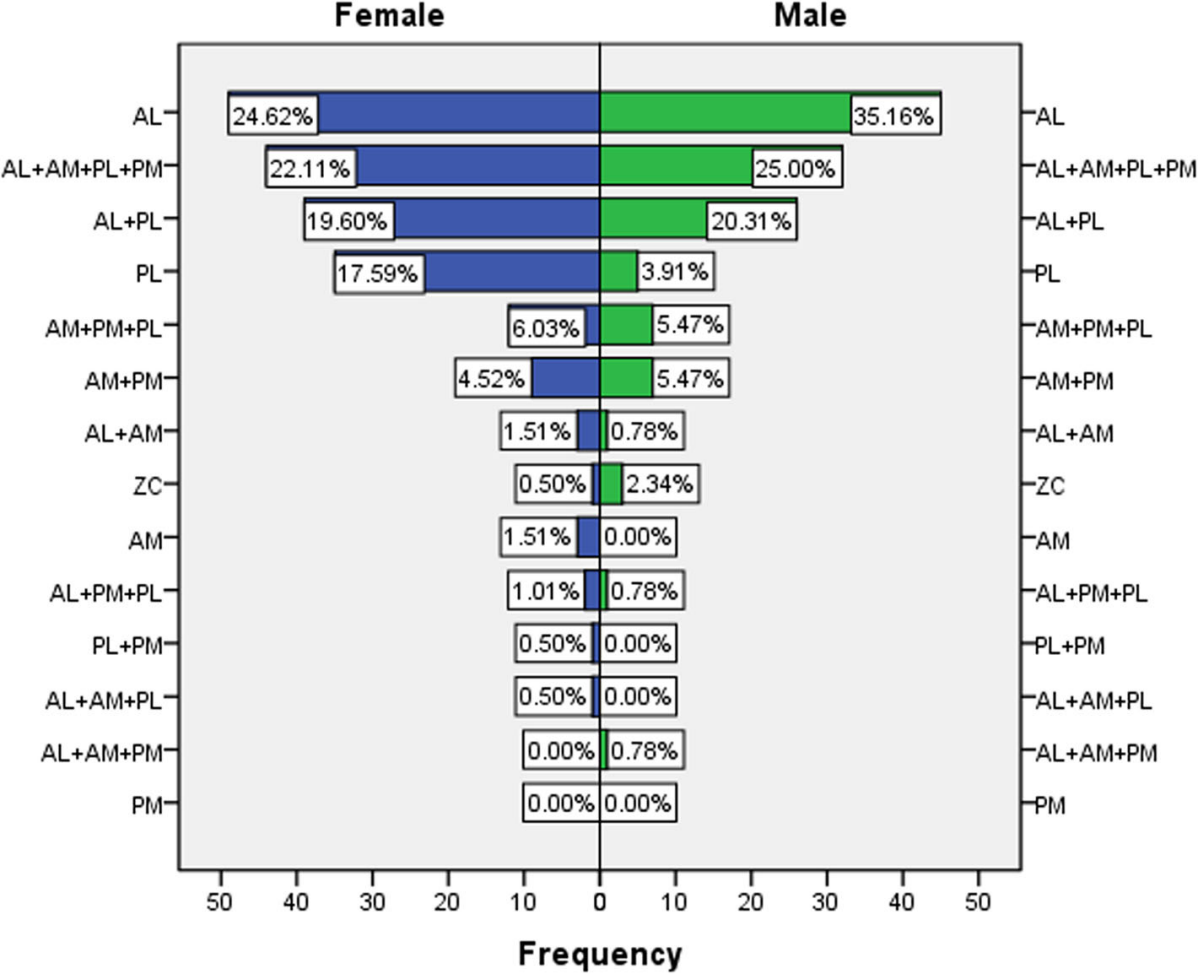
The distribution of fracture patterns based on the TCC in elderly patients between females and males. Compared with males, females had a higher incidence of PL pattern fractures and a lower incidence of AL fractures. The morbidity of complex AL + AM+PL + PM pattern fractures was significantly higher in the male patients

The AL fractures were the most frequent (94, 28.75%), followed by the four quadrants fracture of three columns (AL + AM + PL + PM pattern) (76, 23.24%) and the AL + PL pattern fracture of two columns (65, 19.88%). The least common types were PM (0), PL + PM (1, 0.31%), AL + AM+PM (1, 0.31%), AL + AM+PL (1, 0.31%). The gender difference for TCC is shown in Fig. 5. Compared with males, female patients had a significantly higher proportion of PL pattern (17.59% vs 3.91%, *P* < 0.05).

The posterior column of the tibial plateau was involved in 67.58% of all elderly patients, and the prevalence of PL and PM were 62.69% and 37.92% respectively. The morbidity of PL alone pattern fracture accounted for 12.23%, and the majority occurred in females (35/5). (details are shown in Table 2). There were four cases(3 males, 1 female) of ZC pattern, which were hardly detected from X-ray, only seen from CT slices.

**Table 2:** Distribution of posterior column injuries in elderly patients

Posterior column injuries	Gender		Total
	Female	Male	
PL	35	5	40(12.23%)
PM	0	0	0
PL + PM	1	0	1
AL + PL	39	26	65
AM+PM	9	7	16
AM+PM + PL	12	7	19
AL + PM + PL	2	1	3
AL + AM+PM	0	1	1
AL + AM+PL	1	0	1
AL + AM+PL + PM	44	32	76
PL involved	134 (67.34%)	71 (55.47%)	205 (62.69%)
PM involved	76 (38.19%)	48 (37.5%)	124 (37.92%)
Posterior column involved	142 (71.36%)	79 (61.72%)	221 (67.58%)

*TCC* three-column classification, *AL* anterolateral column, *AM* anteromedial column, *PL* posterolateral sub-column, *PM* posteromedial sub-column

## Discussion

Incidences of TPFs are a complex spectrum of intra-articular fractures around the knee joint which are still of great challenge to treat [13]. Epidemiological and morphological studies of TPFs have been reported to improve the concept and surgical techniques of the treatment [2, 3, 14, 15]. Previously, almost all TPFs-centred studies have analyzed the patients of different age groups as a whole, which possibly confounded the distinctions among age groups [16, 17]. Because of different injury mechanisms and age-related structural variation in bone tissue, the management of TPFs in the elderly population should be very different from that in younger patients and might be more challenging [18–21]. Thus, we isolated an elderly population with TPFs based on age and the epidemiological and morphological characteristics of TPFs in these patients were evaluated and summarized.

In the study, patients aged ≥ 60 years accounted for 23.24% of all consecutively registered patients with TPFs. Among these older patients, females account for 60.86%, which is higher than previously reported in younger patients. This is in accordance with the gender trend of fractures, in which men younger than 50 years have a higher incidence of fractures, but after the age of 50 years, the incidence increases markedly in women and decreases in men [2, 6]. Older female patients often are at high risk of osteoporosis [22, 23]. Decreased bone quality leads to more low-energy traumatic fractures, and this study demonstrated a higher incidence of Schatzker type I, II, and III fractures and one-column fractures in elderly patients than that reported in the literature for younger patients [3, 15]. It is well known that osteoporotic fractures are a frequent and important cause of disability and rising medical costs worldwide; however, researchers on osteoporotic or senile fractures have usually concentrated on the classic fragility fractures of the proximal humerus, distal radius, and proximal femur [23, 24]. Although only 7.34% (24/327) of fractures in this study were due to falling on the ground and can be accurately categorized as fragile fractures, it is obvious that the influence of age or estrogen-related bone structural changes in this group of patients cannot be ignored.

The injury mechanisms may vary between different areas and populations. Among all injury causes of TPFs, traffic accidents range from 35.49 to 54.4%. In elderly patients, more than half of TPFs (57.19%) were due to traffic accidents and E-bike accidents played an important part (32.42%), with 39.62% of them leading to high-energy injuries. Use of E-bikes is a major traffic mode in the plains area of China [25, 26]. Nevertheless, it is not only an important issue in China, because many reports have referred to the global prevalence of the E-bike and its related injuries [27–29]. Tenenbaum et al. [28] reported that 65% of injured E-bike riders sustained orthopedic injuries, and the tibia was the most fractured bone (19.2%) in all E-bike-related fractures. Too fast a speed, carrying passengers, and traffic rule violations are the major reasons for E-bike accidents [29–31]. Importantly, age could be the most important risk factor for E-bike injuries according to a traffic report [32]. In a review according to the Groningen bicycle accident database [33], the average age of injured E-bike riders was 65 years, which is very close to the average age of 66.4 years in our study. Considering that more than one third of TPFs in the elderly were caused by E-bike injuries, related preventive measures would be of benefit to decrease the numbers of these injuries.

According to the CT-based TCC, the pattern of TPFs in elderly patients presented a bipolar model as a whole. The AL fracture alone and the most complicated four quadrants fracture were the two most frequent patterns in the elderly population. Low-energy injury to the knee joint, which might be a valgus injury, always leads to a high frequency of the lateral plateau fracture, including the single-column AL fractures alone or the two-column AL + PL fractures, while the total plateau consisting of three columns and four quadrants might be all related to high-energy trauma due to age-related bone fragility.

In the past decade, posterior column injuries of TPFs have drawn more and more attention. There is growing evidence that posterior tibial plateau fractures affect the functional outcome [17, 34]. The reported incidence of posterior tibial plateau fractures ranges from 28.8 to 70.7% [9, 15]. The results of this study found a clear indication that the posterior column fracture was also associated with high morbidity in elderly patients (67.58%), and the PL sub-column (62.69%) was more often involved than the PM sub-column (37.92%). According to the updated TCC protocol, a ruptured posterolateral wall often needs to be exposed and buttressed [14]. However, because the access to the PL fracture is hindered by the fibula and peroneal nerve to the anterior and by the popliteal neurovascular structures to the posterior, the risk of iatrogenic damage to these adjacent structures during exposure and fixation is high [35–37]. Subsequently, the exposure of the posterolateral articular surface is still insufficient due to the popliteus and strong posterior capsule. To date, although various approaches and fixation patterns have been developed to surgically treat the PL fractures, there are still many defects with these posterolateral approaches [37–40].

Isolated posterior column fractures are uncommon [41, 42]. This type of fracture more frequently occurred in our study compared with our another related study (regardless of age) (12.54% vs 7.8%) [15]. In particular, most isolated posterior column fractures we found were single PL fractures involving female patients (85.37%, 35/41). This single PL fracture was caused by low-energy valgus injury mechanisms in different degrees of knee flexion, which was characterized by a main articular depression or split fragment limited to the posterior half of the lateral column [43]. Yu reported a similar frequency of 11.4% (15/132) in patients with the average age of 53.3 years and indicated that this low-energy posterolateral fracture was highly associated with E-bike injury (8/15 cases) and mostly involved male patients [44]. In our study, we did not investigate further whether this fracture pattern was related to E-bike injury but we found that it mostly occurred in females rather than males in the older population. Another type to be noted is secluded ZC fracture exist in geriatric TPFs. CT scan is necessary when X-ray knee is suspicious.

There are several limitations to this study. First, this is a single-center research study which only represents a regional epidemiology of this population. Second, this remains a qualitative, not quantitative morphological study which is mainly based on the TCC system. Third, there were no clinical features and treatment outcomes for a number of reasons. Different treatment concepts were held by several different treatment teams in our trauma center; therefore, different treatment choices (conservation vs surgery, various surgical approaches and fixation styles, internal fixation vs arthroplasty) might be selected for the TPFs in elderly patients and the follow-up data could not be collected uniformly. Thus, valuable clinical outcome data became difficult to obtain. Further prospective investigations with measurable parameters or refined clinical outcomes are needed for a more accurate assessment and to reveal the full extent of TPFs in the elderly.

## Conclusion

The majority of elderly patients with tibial plateau fractures are females, and the population involved was increasing. Electric-bike accidents are an important cause of injury. Geriatric tibial plateau fractures have unique distribution in classification.

## Abbreviations

AL: Anterolateral
CT: Computerized tomography
E-bike: Electric-bike
PL: Posterolateral
PM: Anteromedial
PM: Posteromedial
TCC: Three-column classification
TPFs: Tibial plateau fractures
ZC: Zero-column

## References

[1] Court-Brown CM, Caesar B (2006). Epidemiology of adult fractures: a review. Injury.

[2] Elsoe R, Larsen P, Nielsen NP, Swenne J, Rasmussen S, Ostgaard SE (2015). Population-based epidemiology of tibial plateau fractures. Orthopedics.

[3] Albuquerque RP, Hara R, Prado J, Schiavo L, Giordano V, do Amaral NP (2013). Epidemiological study on tibial plateau fractures at a level I trauma center. Acta Orto Bras.

[4] Schulak DJ, Gunn DR (1975). Fractures of tibial plateaus. A review of the literature. Clin Orthop Relat Res.

[5] Court-Brown CM, Clement ND, Duckworth AD, Aitken S, Biant LC, McQueen MM (2014). The spectrum of fractures in the elderly. Bone Joint J.

[6] Court-Brown CM, McQueen MM (2016). Global forum: fractures in the elderly. J Bone Joint Surg Am Vol.

[7] Rozell JC, Vemulapalli KC, Gary JL, Donegan DJ (2016). Tibial plateau fractures in elderly patients. Geriatr Orthop Surg Rehabil.

[8] Luo CF, Sun H, Zhang B, Zeng BF (2010). Three-column fixation for complex tibial plateau fractures. J Orthop Trauma.

[9] van den Berg Juriaan, Reul Maike, Nunes Cardozo Menno, Starovoyt Anastasiya, Geusens Eric, Nijs Stefaan, Hoekstra Harm (2017). Functional outcome of intra-articular tibial plateau fractures: the impact of posterior column fractures. International Orthopaedics.

[10] Prat-Fabregat S, Camacho-Carrasco P (2016). Treatment strategy for tibial plateau fractures: an update. EFORT Open Rev.

[11] Sohn HS, Yoon YC, Cho JW, Cho WT, Oh CW, Oh JK (2015). Incidence and fracture morphology of posterolateral fragments in lateral and bicondylar tibial plateau fractures. J Orthop Trauma.

[12] El-Alfy B, Ali KA, El-Ganiney A (2016). Bicondylar tibial plateau fractures involving the posteromedial fragment: morphology based fixation. Acta Orthop Belg.

[13] Reul M, Nijs S, Rommens PM, Hoekstra H (2017). Intra-articulair tibial plateau fractures. Z Orthop Unfall.

[14] Wang Y, Luo C, Zhu Y, Zhai Q, Zhan Y, Qiu W, Xu Y (2016). Updated three-column concept in surgical treatment for tibial plateau fractures - a prospective cohort study of 287 patients. Injury.

[15] Yang G, Zhai Q, Zhu Y, Sun H, Putnis S, Luo C (2013). The incidence of posterior tibial plateau fracture: an investigation of 525 fractures by using a CT-based classification system. Arch Orthop Trauma Surg.

[16] Chen P, Shen H, Wang W, Ni B, Fan Z, Lu H (2016). The morphological features of different Schatzker types of tibial plateau fractures: a three-dimensional computed tomography study. J Orthop Surg Res.

[17] Jiwanlal A, Jeray KJ (2016). Outcome of posterior tibial plateau fixation. J Knee Surg.

[18] Luria S, Liebergall M, Elishoov O, Kandel L, Mattan Y (2005). Osteoporotic tibial plateau fractures: an underestimated cause of knee pain in the elderly. Am J Orthop (Belle Mead, NJ).

[19] Krappinger D, Struve P, Smekal V, Huber B (2008). Severely comminuted bicondylar tibial plateau fractures in geriatric patients: a report of 2 cases treated with open reduction and postoperative external fixation. J Orthop Trauma.

[20] Shimizu T, Sawaguchi T, Sakagoshi D, Goshima K, Shigemoto K, Hatsuchi Y (2016). Geriatric tibial plateau fractures: clinical features and surgical outcomes. J Orthop Sci.

[21] Su EP, Westrich GH, Rana AJ, Kapoor K, Helfet DL (2004). Operative treatment of tibial plateau fractures in patients older than 55 years. Clin Orthop Relat Res.

[22] Alswat KA (2017). Gender disparities in osteoporosis. J Clin Med Res.

[23] Boschitsch EP, Durchschlag E, Dimai HP (2017). Age-related prevalence of osteoporosis and fragility fractures: real-world data from an Austrian menopause and osteoporosis clinic. Climacteric.

[24] Yoo JH, Moon SH, Ha YC, Lee DY, Gong HS, Park SY, Yang KH (2015). Osteoporotic fracture: 2015 position statement of the Korean Society for Bone and Mineral Research. J Bone Metabol.

[25] Zhou SA, Ho AFW, Ong MEH, Liu N, Pek PP, Wang YQ, Jin T, Yan GZ, Han NN, Li G (2017). Electric bicycle-related injuries presenting to a provincial hospital in China: a retrospective study. Medicine.

[26] Li X, Yun Z, Li X, Wang Y, Yang T, Zheng L, Qian J (2017). Orthopedic injury in electric bicycle-related collisions. Traffic Injury Prev.

[27] Siman-Tov M, Radomislensky I, Israel Trauma G, Peleg K (2017). The casualties from electric bike and motorized scooter road accidents. Traffic Inj Prev.

[28] Tenenbaum Shay, Weltsch Daniel, Bariteau Jason T., Givon Adi, Peleg Kobi, Thein Ran (2017). Orthopaedic injuries among electric bicycle users. Injury.

[29] Weber T, Scaramuzza G, Schmitt KU (2014). Evaluation of e-bike accidents in Switzerland. Accid Anal Prev.

[30] Wu C, Yao L, Zhang K (2012). The red-light running behavior of electric bike riders and cyclists at urban intersections in China: an observational study. Accid Anal Prev.

[31] Yang J, Hu Y, Du W, Powis B, Ozanne-Smith J, Liao Y, Li N, Wu M (2014). Unsafe riding practice among electric bikers in Suzhou, China: an observational study. BMJ Open.

[32] Hu F, Lv D, Zhu J, Fang J (2014). Related risk factors for injury severity of e-bike and bicycle crashes in Hefei. Traffic Inj Prev.

[33] Poos H, Lefarth TL, Harbers JS, Wendt KW, El Moumni M, IHF R (2017). E-bikers are more often seriously injured in bicycle accidents: results from the Groningen bicycle accident database. Ned Tijdschr Geneeskd.

[34] Molenaars RJ, Mellema JJ, Doornberg JN, Kloen P (2015). Tibial plateau fracture characteristics: computed tomography mapping of lateral, medial, and bicondylar fractures. J Bone Joint Surg Am.

[35] Sun H, Luo CF, Yang G, Shi HP, Zeng BF (2013). Anatomical evaluation of the modified posterolateral approach for posterolateral tibial plateau fracture. Eur J Orthop Surg Traumatol.

[36] Heidari N, Lidder S, Grechenig W, Tesch NP, Weinberg AM (2013). The risk of injury to the anterior tibial artery in the posterolateral approach to the tibia plateau: a cadaver study. J Orthop Trauma.

[37] Pierrie SN, Harmer LS, Karunakar MA, Angerame MR, Andrews EB, Sample KM, Hsu JR (2016). Limited added value of the posterolateral approach. J Knee Surg.

[38] Solomon LB, Stevenson AW, Lee YC, Baird RP, Howie DW (2013). Posterolateral and anterolateral approaches to unicondylar posterolateral tibial plateau fractures: a comparative study. Injury.

[39] Garner MR, Warner SJ, Lorich DG (2016). Surgical approaches to posterolateral tibial plateau fractures. J Knee Surg.

[40] Cho JW, Samal P, Jeon YS, Oh CW, Oh JK (2016). Rim plating of posterolateral fracture fragments (PLFs) through a modified anterolateral approach in tibial plateau fractures. J Orthop Trauma.

[41] Chang SM, Zheng HP, Li HF, Jia YW, Huang YG, Wang X, Yu GR (2009). Treatment of isolated posterior coronal fracture of the lateral tibial plateau through posterolateral approach for direct exposure and buttress plate fixation. Arch Orthop Trauma Surg.

[42] Tao J, Hang DH, Wang QG, Gao W, Zhu LB, Wu XF, Gao KD (2008). The posterolateral shearing tibial plateau fracture: treatment and results via a modified posterolateral approach. Knee.

[43] Chen HW, Chen CQ, Yi XH (2015). Posterior tibial plateau fracture: a new treatment-oriented classification and surgical management. Int J Clin Exp Med.

[44] Yu GR, Xia J, Zhou JQ, Yang YF (2012). Low-energy fracture of posterolateral tibial plateau: treatment by a posterolateral prone approach. J Trauma Acute Care Surg.

